# Acute hepatitis of unknown origin in children: A combination of factors

**DOI:** 10.3389/fphar.2022.1056385

**Published:** 2022-11-11

**Authors:** Kai Gong, Xianbin Xu, Junjie Yao, Shaoheng Ye, Xia Yu, Huilan Tu, Yan Lan, Yu-chen Fan, Yu Shi

**Affiliations:** ^1^ State Key Laboratory for Diagnosis and Treatment of Infectious Diseases, National Clinical Research Center for Infectious Diseases, Collaborative Innovation Center for Diagnosis and Treatment of Infectious Diseases, The First Affiliated Hospital, College of Medicine, Zhejiang University, Hangzhou, Zhejiang, China; ^2^ Department of Hepatology, Qilu Hospital, Cheeloo College of Medicine, Shandong University, Jinan, Shandong, China

**Keywords:** acute hepatitis, adenovirus, SARS-CoV-2, multisystem inflammatory syndrome in children, adeno-associated virus 2, immunodeficiency

## Abstract

On 5 April 2022, the World Health Organization was notified of 10 cases of severe acute hepatitis of unknown etiology in children under 10 years of age in the United Kingdom. Although the exact cause of a proportion of pediatric acute hepatitis and acute liver failure cases was unclear, the above event has caused widespread concern worldwide. As of 14 September 2022, approximately 1,296 probable cases of acute hepatitis of unknown etiology have been reported from 37 countries/regions, of which approximately 55 required or received liver transplantation and 29 died. Although the etiology of acute hepatitis of unknown origin in children remains unclear, many hypotheses have been proposed about the disease. Instead of individual factors such as “adenovirus infection,” “SARS-CoV-2 related,” and “Adeno-associated virus 2 with helper virus coinfection,” it is more likely due to a combination of factors. Accordingly, there is an urgent need for more data and research to clarify the disease etiology. This review aims to provide a historical perspective of acute hepatitis of unknown etiology in children in the past decades and summarize the current hypothesis and evidence on this emerging disease.

## Graphical Abstract

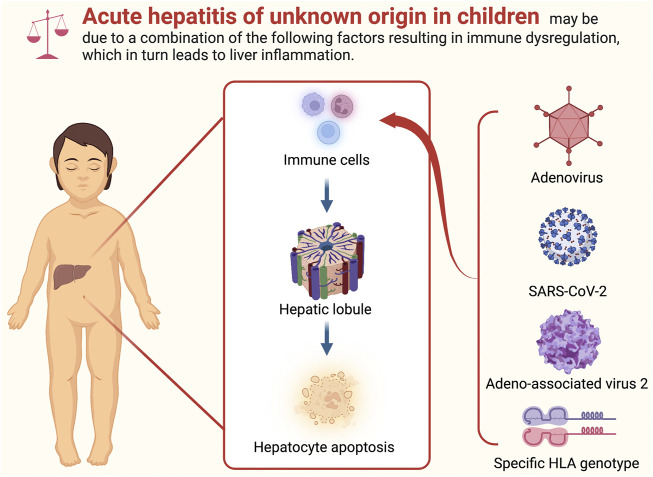

## 1 Introduction

The purpose of this article is to give a framework and reference for understanding the etiology of acute hepatitis of unknown origin in children, taking into consideration newly published relevant literature and information data.

On 31 March 2022, severe acute hepatitis of unknown origin was reported for the first time among children in Scotland for causes other than common hepatitis A-E virus infection (Marsh et al., 2022). Since then, similar cases have been reported in many countries or regions worldwide, and the number of cases has rapidly increased, causing widespread concern. Acute hepatitis is caused by various pathogenic factors invading the liver, damaging hepatocytes and liver function, and resulting in severe clinical symptoms. At first, patients experience fatigue, fever and loss of appetite, and in more severe cases, nausea, vomiting or tea-colored urine occur, followed by jaundice. The disease course does not exceed half a year. These common pathogenic factors are viruses, bacteria, parasites, chemical poisons, drugs and poisons, alcohol, *etc.*

This article aims to provide a framework and reference for understanding the etiology of acute hepatitis of unknown origin in children, taking into consideration newly published relevant literature and information data.

## 2 Methods

We sought to conduct a review of the literature to better understand acute hepatitis of unknown origin in children. PubMed database was retrieved using the search terms “pediatrics” or “children” and “hepatitis” or “liver failure” to identify relevant studies published from inception until 14 September 2022. The full text of relevant titles and abstracts was then reviewed for inclusion. In addition, citations and references of included studies were searched to identify any additional studies. English-language peer-reviewed and non-peer-reviewed studies describing acute hepatitis in children, pediatric acute liver failure (PALF), adenovirus, SARS-CoV-2, adeno-associated virus 2, and autoimmune hepatitis were included.

## 3 Disease background

### 3.1 Determinate and indeterminate pediatric acute hepatitis and liver failure

**FIGURE 1:**
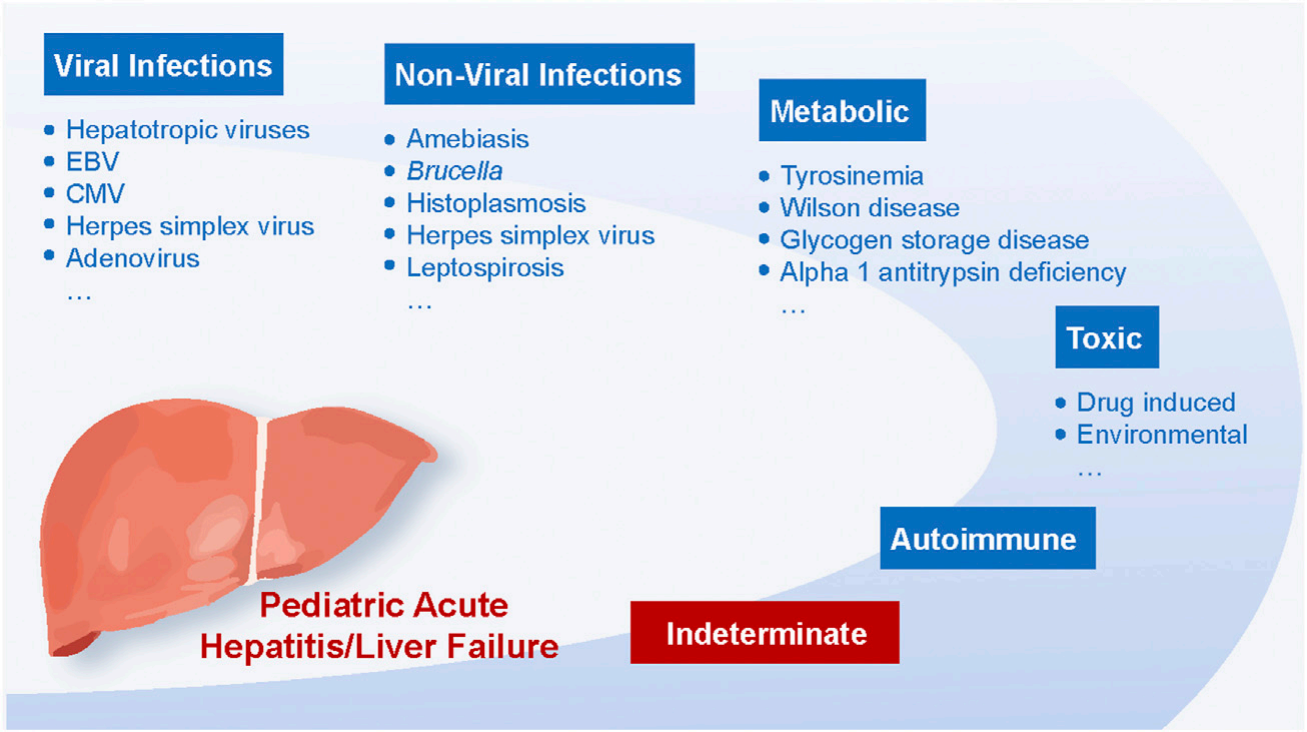
Determinate pediatric acute hepatitis and liver failure.

Over the past few decades, acute hepatitis and liver failure in children have become major public health issues worldwide (Daniels et al., 2009). Infectious, toxic, autoimmune, and metabolic mechanisms are the most common causes (Figure 1) (Devictor et al., 2011).

Most cases can be attributed to viral infections, with hepatotropic viruses being the primary culprit (Daniels et al., 2009; Zeng et al., 2021). Globally, acute hepatitis A virus (HAV) infection has long been considered the most prevalent cause of acute hepatitis in children (Rahman et al., 2022). However, its prevalence varies by geographical area and vaccination rates (Daniels et al., 2009; Zeng et al., 2021). Studies in the United Kingdom and India indicated that HAV infection accounts for 39% and 48.6% of acute hepatitis in hospitalized children, respectively (Braccio et al., 2017; Sood et al., 2019). Since the beginning of the century, routine childhood immunization has been promoted continuously in numerous nations, including the United States and Europe. In this regard, from 1995 to 2007, the incidence of acute hepatitis A in the United States population decreased by 92%, especially for children under 15 years old (Daniels et al., 2009). In addition to HAV, sporadic and epidemic infections of the Hepatitis E virus (HEV) represent a leading cause of acute viral hepatitis in children, primarily in developing nations (Verghese and Robinson, 2014; Fischler et al., 2016). HEV seroprevalence in children increases with age (Hodges et al., 1998) and is related to residence and socioeconomic status (Arankalle et al., 2001). Two early studies with small sample sizes from Egypt showed that acute sporadic hepatitis E accounted for 22.2% (under 11 years old) (el-Zimaity et al., 1993) and 12% (under 13 years old) (Hyams et al., 1992) of children admitted to hospital for acute hepatitis.

Besides hepatotropic pathogens, other viruses such as Epstein-Barr virus (EBV), cytomegalovirus (CMV), herpes simplex virus, adenovirus, and coxsackievirus can also cause acute liver failure in children, especially in those with immunodeficiency disorders (Gallegos-Orozco and Rakela-Brödner, 2010; Tsunoda et al., 2017). Real-time PCR and serological testing can assist in the detection of non-hepatitis virus-related acute hepatitis in children. A recent study investigated the incidence of CMV and EBV in children hospitalized with acute liver failure. Of the children excluded from hepatitis virus A, B, and C infection, 18% and 9% were positive for both DNA and IgM assay of CMV and EBV, respectively (Tsunoda et al., 2017). Another prospective study proposed that human herpesvirus-6 could cause acute liver failure in children (Chevret et al., 2008).

Other non-viral infections can potentially cause acute hepatitis in children, including amebiasis (Gupta et al., 2022), *Brucella* (Hassouneh et al., 2019), histoplasmosis, leptospirosis (Aygün et al., 2016), *etc.* A new study suggested that hepatitis was the most common laboratory abnormality in children with brucellosis (nearly three-quarters) (Hassouneh et al., 2019). In recent years, pediatric autoimmune hepatitis (AIH) has been increasingly reported (Mack et al., 2020). In the United States, the prevalence of AIH is 3 per 100,000 children, and the estimated annual incidence per 100,000 children is 0.4, comparable to Canada (0.23) (Deneau et al., 2013; Jiménez-Rivera et al., 2015).

In addition, drugs represent another potential cause of acute hepatitis. In developed countries, acetaminophen is the leading cause of drug-induced liver injury in children, followed by medications such as anti-tuberculosis and anti-epileptic agents (Squires et al., 2006). As for newborns and infants, acute liver failure is most commonly caused by inherited metabolic diseases (Durand et al., 2001), including Wilson disease, alpha-1 antitrypsin deficiency, tyrosinemia, glycogen storage disease, *etc.*

Although in-depth and comprehensive research has been conducted on the etiology of pediatric acute hepatitis and acute liver failure along with the continuous promotion of unprecedented metabolic and viral screening, a definite cause cannot be found in a substantial portion of children (Aydoğdu et al., 2003; Squires et al., 2006; Black, 2009; Narkewicz et al., 2009). When a specific diagnosis is not established in PALF, these children are categorized as indeterminate (iPALF) (Alonso et al., 2017). The Pediatric Acute Liver Failure study group, composed of centers from the United States, Canada, and the United Kingdom, released the initial data of 348 cases in 2006 (Squires et al., 2006). The outcomes of this prospective multicenter research revealed that for nearly half of children with pediatric acute liver failure, a definitive diagnosis could not be determined, consistent with the subsequent results published by this study group in 2008 and the conclusions of studies from Turkey and Germany (Aydoğdu et al., 2003; Squires et al., 2006; Narkewicz et al., 2009; Kathemann et al., 2015). Besides, children with ambiguous diagnoses were younger and more likely to receive liver transplantation after admission than children with a definitive diagnosis (Liu et al., 2006; Narkewicz et al., 2009). In subsequent studies, Narkewicz et al. evaluated the diagnostic mode of 329 children who were eventually diagnosed with indeterminate acute liver failure between 1999 and 2008. The results showed inadequate screening for metabolic and autoimmune acute liver failure causes. Furthermore, an efficient diagnostic evaluation pattern for pediatric acute liver failure based on age and clinical manifestations should be established to help pediatricians formulate timely and effective treatment strategies (Narkewicz et al., 2009). Mechanisms driving injury of iPALF have been postulated to be secondary to overwhelming inflammatory responses and immune dysregulation (Alonso et al., 2017). Recent efforts to further characterize iPALF have identified a subset of patients with unusual liver histology, including a dense CD103+CD8^+^ T-cell infiltrate, further suggesting liver injury is immune-mediated (Squires et al., 2022).

## 4 Epidemiology

**FIGURE 2:**
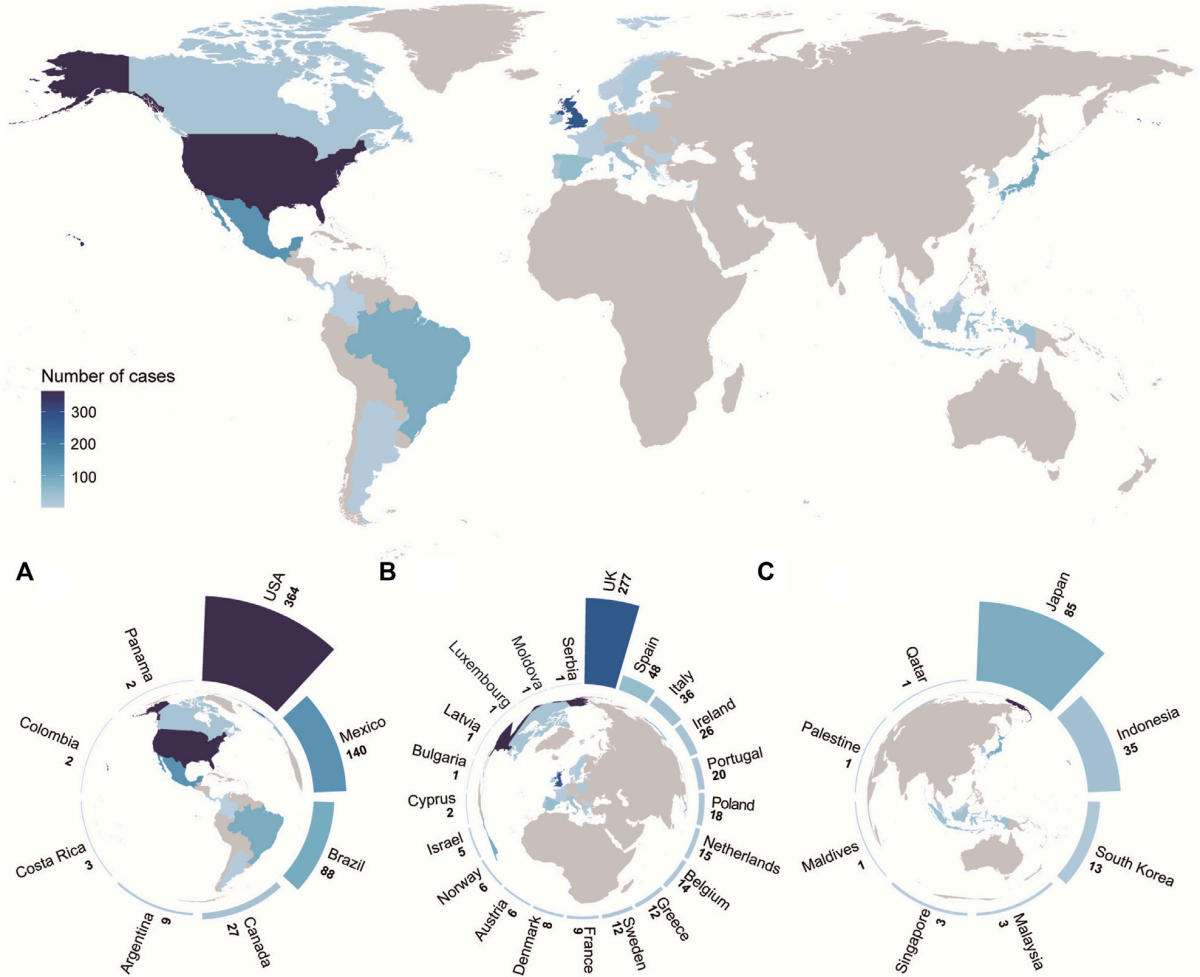
World distribution of possible cases, as of 14 September 2022 (*n* = 1,296) **(A)** Distribution of possible cases in the Americas. **(B)** Distribution of possible cases in Europe. **(C)** Distribution of possible cases in Asia. The above four figures share the same legend. Further details are provided in Table 1.

As of 14 September 2022, approximately 1,296 probable cases of acute hepatitis of unknown etiology have been reported from 37 countries/regions, of which approximately 55 required or received liver transplantation and 29 died. The highest incidence was in the United States (*n* = 364), followed by the United Kingdom (*n* = 277), Mexico (*n* = 140), Brazil (*n* = 88), and Japan (*n* = 85). Based on the available data, 12% (80/660) of the tested cases were positive for severe acute respiratory syndrome coronavirus 2 (SARS-CoV-2) polymerase chain reaction (PCR), 46.7% (372/797) were positive for adenovirus PCR, and 75.2% (76/101) of the detected adenovirus cases were type 41 (World Health Organization, 2022b; European Centre for Disease Prevention and Control, 2022; Government of Canada, 2022). More details are shown in Table 1 and Figure 2.

**TABLE 1:** Summary of possible cases reported by countries/regions as of 14 September 2022.

	Country/Region	Number of cases	Required liver transplant	Deaths	SARS-CoV-2 positive by PCR	Adenovirus positive by PCR	Adenovirus type 41
Americas	Argentina	9	1	—	0/3	2/3	1/3
	Brazil	88	—	—	—	—	—
	Canada	27	3	0	3/20	3/18	0/1
	Colombia	2	0	0	0/2	1/2	0/2
	Costa Rica	3	—	—	—	3/3	—
	Mexico	140	0	1	—	—	—
	Panama	2	0	—	—	—	—
	United States of America	364	22	13	15/197	135/299	13/22
Europe	Austria	6	0	0	1/3	0/3	—
	Belgium	14	0	0	3/14	2/7	—
	Bulgaria	1	0	0	0/1	0/1	—
	Cyprus	2	0	0	0/1	1/2	0/1
	Denmark	8	0	0	2/8	0/7	0/1
	France	9	0	0	0/8	4/6	0/1
	Greece	12	0	0	0/9	2/10	—
	Ireland	26	2	1	0/8	9/16	—
	Israel	5	0	0	0/2	1/2	—
	Italy	36	1	0	2/19	11/25	—
	Latvia	1	0	0	—	1/1	—
	Luxembourg	1	0	0	0/1	0/1	—
	Moldova	1	0	0	0/1	0/1	—
	Netherlands	15	4	0	1/4	4/9	—
	Norway	6	0	0	2/5	2/5	2/2
	Poland	18	0	0	0/2	2/5	—
	Portugal	20	0	0	4/14	2/13	0/1
	Serbia	1	1	0	0/1	1/1	—
	Spain	48	3	2	3/29	5/28	1/2
	Sweden	12	2	0	2/9	4/9	—
	United Kingdom	277	15	0	36/237	170/258	59/62
Asia	Indonesia	35	0	11	—	—	—
	Japan	85	0	0	5/59	5/58	—
	Malaysia	3	—	—	—	—	—
	Maldives	1	0	—	—	—	—
	Palestine	1	0	1	—	—	—
	Qatar	1	—	—	—	1/1	—
	Singapore	3	0	0	1/3	1/3	0/3
	South Korea	13	1	0	—	—	—
Total		1,296	55	29	80/660 (12.1%)	372/797 (46.7%)	76/101 (75.2%)

According to ECDC and The European Surveillance System (TESSy), the majority (76.2%) of cases were 5 years old or younger, with no significant difference in gender (European Centre for Disease Prevention and Control, 2022).

## 5 Clinical features

The clinical manifestations are characterized by an acute onset of clinical symptoms, including weakness and poor appetite, nausea, vomiting, diarrhea, abdominal pain, and other gastrointestinal symptoms, followed by jaundice. Moreover, children may present with pale stools, liver enlargement, fever, respiratory symptoms, or splenomegaly. In rare cases, the disease may progress to acute liver failure within a short period, with progressive worsening of jaundice and hepatic encephalopathy. Rapid disease progression may require liver transplantation and even lead to death in severe cases.

According to the description in Technical Brief 4 published by The UK Health Security Agency (UKHSA), most patients present with jaundice (69.2%), followed by vomiting (57.9%), and pale stools (40.5%). Patients may also present with gastrointestinal symptoms, including diarrhea (41.5%), abdominal pain (38.5%), and nausea (26.2%). In addition, lethargy (47.7%), fever (23.1%) and some rare respiratory symptoms (17.9%) have been reported (UK Health Security Agency, 2022c).

## 6 Case definitions

Currently, different organizations/countries use different case definitions of confirmed, probable, and epi-linked. The WHO, United Kingdom, Scotland, the European Union, the United States, and Canada categorize cases as confirmed, probable, and epi-linked, using different case definitions based on case age, reporting window, and other etiologic specifications and exclusions. Table 2 summarizes different case definitions in different organizations (World Health Organization, 2022a; UK Health Security Agency, 2022b; Centre for Disease Prevention and Control, 2022; European Centre for Disease Prevention and Control, 2022; Government of Manitoba, 2022).

**TABLE 2:** The different case definitions in the different organizations.

Organization	Case definitions			
	Confirmed	Probable	Epi-linked	Addition
World Health Organization	N/A	(1) Age ≤16	(1) Any age who is a close contact of a probable case	Discarded: Cases with other explanations for their clinical presentation are discarded.
		(2) since 1 October 2021		
		(3) acute hepatitis (non hep A-E)	(2) since 1 October 2021	Pending classification: Hepatitis A-E serology results are awaited, but other criteria met.
		(4) AST or ALT >500 IU/L	(3) acute hepatitis (non hep A-E)	
UK Health Security Agency (England, Wales, Northern Ireland)	(1) Age ≤10	(1) Age 11-15	(1) a close contact of a confirmed case.	Cases should be reported based on clinical judgement if some hepatitis A-E virus results are awaited, or if there is an acute on chronic hepatic presentation with a metabolic, inherited/genetic, congenital, mechanical or other underlying cause (in Wales, this also excludes known critical illness).
	(2) since 1 January 2022	(2) since 1 January 2022	(2) since 1 January 2022	
	(3) acute hepatitis which is not due to hepatitis A-E viruses, or an expected presentation of metabolic, inherited or genetic, congenital or mechanical cause	(3) acute hepatitis which is not due to hepatitis A-E viruses or an expected presentation of metabolic, inherited or genetic, congenital or mechanical cause	(3) acute hepatitis (non-hepatitis A-E) (A person who is epi-linked but also meets the confirmed or possible case definition will be recorded as a confirmed or possible case and their epi-link noted in their record. This prevents double-counting of cases).	Pending classification: Hepatitis A-E serology results are awaited, but other criteria met.
	(4) AST or ALT >500 IU/L	(4) AST or ALT >500 IU/L		
UK Health Security Agency (Scotland)	(1) Age ≤10 or a contact of any age of a confirmed case	N/A	N/A	Excluding hepatitis A-E, CMV and EBV.
	(2) since 1 January 2022			
	acute hepatitis without any known cause			
	(3) AST or ALT >500 IU/L			
European Centre for Disease Prevention and Control	N/A	(1) Age ≤16	(1) Any age who is a close contact of a probable case	Discarded: A subject previously classified as case, that following further investigations did not meet the case definition criteria.
		(2) since 1 October 2021		
		(3) acute hepatitis (non-hepatitis viruses A, B, C, D, E)	(2) since 1 October 2021	Cases of hepatitis with known aetiology such those due to specific infectious diseases, drug toxicity, metabolic hereditary, or autoimmune disorders should not be reported under this protocol.
		(4) AST or ALT >500 IU/L	(3) acute hepatitis (non-hepatitis viruses A, B, C, D, E)	
U.S. Centers for Disease Control and Prevention	N/A	(1) Age <10	N/A	N/A
		(2) since October 1 2021		
		(3) hepatitis with an unknown etiology (with or without any adenovirus testing results, independent of the results)		
		(4) AST or ALT >500 IU/L		
The Public Health Agency of Canada	N/A	(1) Age ≤16	N/A	If hepatitis virus D or E serology results are pending or test was not done, but other criteria met, these can be reported as probable cases.
		(2) since 1 October 2021		
		(3) severe acute hepatitis requiring hospitalization		
		(4) AST or ALT >500 IU/L		
		(5) excluding hepatitis caused or attributed to a hepatitis virus (A, B, C, D, E) or a known or expected presentation of a drug or medication; a genetic, congenital, or metabolic condition; an oncologic, vascular, or ischemia related condition; or an acute worsening of chronic hepatitis		

## 7 Diagnosis

Patients who meet one of the definitions of probable cases and epi-linked cases should be comprehensively investigated, including the collection of epidemiological and family history. Routine laboratory tests should be performed, including blood cell analysis, biochemical tests, coagulation tests, plasma ammonia, inflammatory markers, and abdominal imaging. All instances that meet the case criteria should undergo testing of whole blood, serum, urine, feces, respiratory samples, and liver biopsy (if available). Laboratory tests for infectious and non-infectious causes are shown in Table 3.

**TABLE 3:** Laboratory testing in acute hepatitis of unknown origin in children.

Cause	Sample type	Recommended test
Virus	Whole blood	• NAAT: SARS-CoV-2, Adenovirus, enterovirus, CMV, EBV, HSV-1, HSV-2, HHV6, HHV7, VZV, parvovirus B19
		• Serology: CMV, EBV, SARS-CoV-2 (S&N proteins), VZV, adenovirus, parvovirus, rubella virus
	Throat swab	• NAAT: Respiratory virus panel (including adenovirus, bocavirus, enterovirus, influenza, parainfluenza, rhinovirus, RSV, SARS-CoV-2)
	Stool	• NAAT: Adenovirus, astrovirus, enterovirus, norovirus, rotavirus, sapovirus, CMV, HPeV
Bacteria	Whole blood	• Standard culture for bacteria
	Throat swab	• Standard bacterial panel, including *Streptococcus* group A
	Stool	• Standard bacterial stool pathogen panel, including *Salmonella* sp
	Urine	• Standard bacterial urine culture
Fungi	Whole blood	• Standard culture for fungi
AIH	Whole blood	• ANA, AMA, LKM, LC, ANCA, IgG subclass
	Liver tissue	• HE staining, immunocytochemistry: IgG4, HLA genotype
IMLD	Whole blood	• Serum copper blue protein, serum iron, arterial blood gas, anion gap, blood amino acid
	Urine	• Urinary organic acid analysis
Toxic	Whole blood/Urine	• Local investigations according to medical history and geography

NAAT, nucleic acid amplification test; SARS-CoV-2, severe Acute Respiratory Syndrome Coronavirus 2; CMV, cytomegalovirus; EBV, Epstein-Barr virus; HSV, herpes simplex virus; HHV, human herpus virus; VZV, varicella zoster virus; RSV, respiratory syncytial virus; HPeV, human parechovirus; AIH, autoimmune hepatitis; ANA, antinuclear antibody; AMA, anti-mitochondrial antibody; LKM, liver kidney microsome, LC, liver cytosol antigen; ANCA, antineutrophil cytoplasmic antibody; HE, hematoxylin-eosin; HLA, human leukocyte antigen; IMLD, inherited metabolic liver disease.

## 8 Working hypotheses on possible etiology

The primary hypotheses about the cause of acute hepatitis in children are as follows:

(1) Adenovirus infection (e.g., abnormal susceptibility or host response due to lack of exposure during the COVID-19 pandemic.; a large-scale outbreak of normal adenovirus infection; coinfection with or priming by another pathogen)
(2) SARS-CoV-2 related (e.g., sequelae from SARS-CoV-2 infection; a postinfectious SARS-CoV-2 syndrome (similar to the multisystem inflammatory syndrome in children); as superantigens; undescribed sign or symptom of a recently emerged variants of concern (VOC) (i.e., Omicron))
(3) Adeno-associated virus 2 with helper virus coinfection (e.g., adenovirus; human herpesvirus 6B)
(4) Immunodeficiency (e.g., a specific HLA genotype: DRB1*04:01)
(5) A novel pathogen not detectable with current diagnostics either acting alone or as a coinfection
(6) A non-infectious cause (e.g., toxin, drug, or environmental exposure)

### 8.1 Adenovirus infection

By far, adenovirus is most frequently identified in test samples. As of 28 July 2022, adenovirus has been found in 65.9% (170/258) of cases investigated in the United Kingdom (UK Health Security Agency, 2022c). Type 41 represented 95.2% (59/62) of these adenovirus-positive cases and was found in several cases reported in the United States (Baker et al., 2022; UK Health Security Agency, 2022c).

Recently, the UK Health Security Agency has documented an increase in adenovirus-positive stool samples from children aged 1–4 years compared to pre-pandemic levels, as well as a rise in the occurrence of hepatitis among children aged 1 to 4 (UK Health Security Agency, 2022b). However, on June 14, the CDC released a preliminary survey highlighting that current data do not indicate that pediatric hepatitis or adenovirus types 40/41 are above pre-pandemic baseline levels of COVID-19 (Kambhampati et al., 2022).

Although adenovirus infection is currently the most widely accepted hypothesis, it cannot fully explain the severity of clinical symptoms as the underlying cause. Biopsies of liver specimens from the United Kingdom, Israel, and Alabama, United States, did not reveal adenovirus inclusions, and the adenovirus load of affected children was generally low (Baker et al., 2022; UK Health Security Agency, 2022b; Marsh et al., 2022). Much controversy surrounds the ability of the “adenovirus hypothesis” to explain the pathogenesis of acute hepatitis of unknown origin in children.

It is well-established that adenovirus infections are prevalent in children. Indeed, children’s tonsils and adenoids are common host organs for adenoviruses, with a reported positivity rate of 76% for adenovirus PCR (Jaggi et al., 2013). About 30% of immunocompetent children develop persistent or resident infections after the acute phase of adenovirus infection. Such persistent infections are common in mucosal lymphocytes and are more common in young children, fading away with age (Garnett et al., 2002; Kosulin, 2019).

Then, during systemic immunocompromise, mucosal-site adenoviruses (as well as various other viruses) have the potential to enter the bloodstream and reactivate, eventually presenting as disseminated infection. Current evidence suggests that acute liver injury and liver failure, regardless of the cause, represent a state of immunodeficiency. However, whether liver damage or adenovirus viremia is the cause remains unclear.

Moreover, almost all previously reported cases of adenoviral hepatitis involved immunodeficiency. In this respect, adenoviral inclusion bodies were detected in all liver biopsies of adenoviral hepatitis. Few cases of acute severe hepatitis or liver failure due to adenovirus infection have been reported in immunocompetent children. However, the serotype human adenovirus (HAdV) 41 has not been linked to hepatitis or liver failure (Rocholl et al., 2004; Ozbay Hoşnut et al., 2008; Lynch and Kajon, 2016; Kang, 2017).

Although adenovirus is infrequently related to fulminant liver failure in immunocompetent children, other factors may enhance susceptibility; hence, adenovirus remains a potential contributing factor in addition to other factors such as abnormal susceptibility or host response.

Interestingly, it has been reported that the outbreak control during the 2019 coronavirus epidemic may have reduced young children’s exposure to common pathogens, leading to the development of immune systems different from their peers before the 2019 coronavirus epidemic. When young children are subsequently infected with common pathogens such as adenovirus, the immune response or disease manifestations differ from their peers. According to the UKHSA, adenovirus, enterovirus, human metapneumovirus, rhinovirus, and norovirus have been significantly overrepresented among children under 10 years old since the end of 2021. Respiratory syncytial viruses have been reported since late summer 2021, possibly due to behavioral alterations and population vulnerability following the pandemic’s low incidence (UK Health Security Agency, 2022b).

### 8.2 SARS-CoV-2 related

Up to now, more than 10% of positive SARS-CoV-2 cases have been detected by PCR, while the positive rate of SARS-CoV-2 serological tests is about 70%. Serological tests are underway to explore previous infections further. (World Health Organization, 2022a; European Centre for Disease Prevention and Control, 2022). Most patients were only tested for SARS-CoV-2 PCR but not for SARS-CoV-2 serology. Notably, SARS-CoV-2 PCR can only determine if the child was infected with SARS-CoV-2 then, but serological testing is indicated to assess whether the child has been infected with SARS-CoV-2 in the past.

SARS-CoV-2 can result in liver function impairment in COVID-19 patients. Recent research by Ellen K. Kendall et al. revealed that children infected with COVID-19 had a two-fold higher risk of liver injury than children infected with other respiratory infections. This finding implies that COVID-19 causes acute and chronic hepatic complications in pediatric patients (Kendall et al., 2022).

Recently, Shiri Cooper et al. analyzed the clinical presentation and laboratory test results of five hospitalized children who recovered from COVID-19 and subsequently exhibited liver injury. It has been suggested that SARS-CoV-2 infection is more likely to cause acute hepatitis of unknown etiology in children. The two patterns of long-term hepatic manifestations of COVID-19 in children include acute liver failure and acute hepatitis with cholestasis (Cooper et al., 2022).

In 2020, Yijin Wang et al. studied 156 COVID-19 patients from two designated centers in China and compared the clinical characteristics of patients with and without increased aminotransferases. The findings indicate that SARS-CoV-2 infection of the liver is a major contributor to the impairment of liver function in COVID-19 patients. However, the median levels of ALT were 50 U/L and 19 U/L, and AST levels were 45.5 U/L and 19 U/L in the abnormal and normal transaminase groups, respectively (Wang et al., 2020). Even the transaminase values in the abnormal transaminase group were much lower than those in the case definition.

However, two independent research groups in Glasgow and London, UK, have recently found no direct association between acute hepatitis of unknown origin in children and SARS-CoV-2 infection. The prevalence of SARS-CoV-2 antibodies did not differ between cases and age-matched controls. Nevertheless, they could not completely rule out an immune-mediated post-COVID-19 phenomenon in susceptible children (Ho et al., 2022; Morfopoulou et al., 2022).

#### 8.2.1 Vaccine

It has been suggested that vaccination with the SARS-CoV-2 mRNA vaccine can trigger T-cell-dominant autoimmune hepatitis mediated by vaccination-induced antigen-specific tissue-resident immunity necessitating systemic immunosuppression (Boettler et al., 2022). Cases of SARS-CoV-2 vaccine-induced autoimmune hepatitis have also been reported prior to October 2021 (Bril et al., 2021).

However, there is no proof to substantiate that acute hepatitis of unknown origin in children is associated with the side effects of SARS-CoV-2 vaccines. According to the WHO, the hypothesis that these cases result from the side effects of SARS-CoV-2 vaccines is not supported, given that most afflicted children were not vaccinated (World Health Organization, 2022a).

#### 8.2.2 MIS-C and SARS-CoV-2 as superantigens

Multisystem inflammatory syndrome in children (MIS-C) often occurs 6–8 weeks after SARS-CoV-2 infection. MIS-C may manifest clinically as persistent fever, gastrointestinal symptoms (abdominal discomfort, vomiting, diarrhea), rash, and conjunctivitis. Patients often present with three to 5 days of fever, followed by the onset of shock and/or multisystem involvement.

Acute hepatitis has been associated with multisystem inflammatory syndrome in children. Amanda Cantor et al. concluded from a small retrospective cohort study that acute hepatitis is an outstanding manifestation of MIS-C (Cantor et al., 2020).

Recently, Petter Brodin and Moshe Arditi have proposed a novel potential mechanism for acute hepatitis of unknown origin in children - superantigens-mediated, adenovirus-induced immune activation by SARS-CoV-2 infection, representing an alternative manifestation of MIS-C (Brodin and Arditi, 2022).

SARS-CoV-2 infection in children represents a viral reservoir in the body (Brodin, 2022). Specifically, the persistence of SARS-CoV-2 in the gastrointestinal tract of children can result in the sustained release of viral proteins from intestinal epithelial cells, thus causing immune activation. The superantigen motif may mediate this recurrent immune activation in the spike protein of SARS-CoV-2, which, similar to staphylococcal enterotoxin B, can trigger widespread and nonspecific t-cell activation (Cheng et al., 2020). This superantigen-mediated activation of immune cells has been recognized as a mechanism of MIS-C.

Accordingly, Petter Brodin and Moshe Arditi speculated that recent cases of acute hepatitis in children may have resulted from the emergence of a viral reservoir in the gut following infection with SARS-CoV-2, followed by adenovirus infection and the dysregulation of the body’s immune response in response to a combination of triggers from the adenovirus and SARS-CoV-2 S protein, leading to the development of acute hepatitis. Importantly, immunomodulatory therapy should be considered for acute hepatitis of unknown origin in children if evidence of superantigen-mediated immune activation is available (Brodin and Arditi, 2022).

A cohort study by Rita Robin involving 113 persons found that a small proportion of COVID-19 patients still had viral RNA in their feces after testing negative for respiratory samples. Interestingly, the condition reportedly persists for about 4% of patients for 7 months or longer, and these patients are more likely to experience persistent gastrointestinal symptoms (Rubin, 2022). These findings add to the growing body of evidence that SARS-CoV-2 invades the gut and provide circumstantial evidence for the speculations of Petter Brodin and Moshe Ardit.

Nonetheless, this theory does not account for the immunopathological liver damage in acute hepatitis of unknown origin in children, whereas MIS-C leads to inflammatory damage in multiple organ systems. Moreover, only a tiny proportion of children with unexplained hepatitis in children present with fever, whereas all MIS-C have fever >3 days.

During the epidemic of the delta variant in India (April 2021 to July 2021), Covid-19-associated hepatitis was reported in 37 children 2–6 weeks after infection with SARS-CoV-2, which was named COVID-19 Associated Hepatitis in Children (CAH-C), and these cases were similar to recent cases of acute hepatitis of unknown origin in children, with a median AST 942.45 (301.67, 2002.05) U/L and a median ALT of 1,326.25 (492.12, 2124.92) U/L, and no jaundice. Radha Kanta Ratho et al. found that CAH-C cases differed from MIS-C, which lacked the significantly elevated inflammatory markers or systemic disturbances seen with MIS-C during all testing periods. The more severe form of MIS-C is characterized by polyclonal T-cell activation, whereas the milder form of CAH-C involves polyclonal B-cell activation (Ratho et al., 2022).

Recently, Keith Sacco et al. described different immunological features in pediatric COVID-19 (pCOVID-19) and MIS-C. Pediatric COVID-19 is characterized by a substantial type I interferon (IFN) response, whereas MIS-C is related to type II IFN-dependent and NF-κB-dependent characteristics, activation of the extracellular matrix, and higher amounts of circulating SARS-CoV-2 stinger protein. However, the only strains included in the study were the ancestral Wuhan strain, the B1.177 (European lineage) variant and the 1.1.7 (Alpha) variant. Thus, the effect of Delta and Omicron variants on the innate and adaptive immune responses in children with pCOVID-19 and MIS-C requires further study (Sacco et al., 2022).

#### 8.2.3 The recent emergence of VOCs

Notably, given that most of the above emergence studies were conducted before the emergence of the Omicron variant (and even the Delta variant), there are big gaps in our knowledge of new symptoms or sequelae associated with the recent emergence of VOCs.

On April 26, a seroepidemiological study published by the United States CDC showed that as of February 2022, about 75% of children and adolescents had serological evidence of past SARS-CoV-2 infection. Since December 2021, about one-third of children and adolescents have new seropositive (Clarke et al., 2022).

Hiroshi Nishiura et al. analyzed the cumulative incidence of COVID-19 from 1 December 2021, to 27 April 2022, in 38 countries of the Organization for Economic Cooperation and Development (OECD) and Romania. Using Welch’s analysis of variance (ANOVA), it was found that countries with hepatitis cases were more likely to experience more Omicron cases, suggesting that prior exposure to the Omicron variant (B.1.1.529) might enhance children’s risk of severe hepatitis, highlighting the importance of cofactor research (Nishiura et al., 2022).

However, data from the whole population may not be representative of the child population. Recently Yi H et al. collected children-specific population data from the ECDC and analyzed the link between Omicron infection in children and the emergence of acute hepatitis of unknown etiology. A positive correlation between cumulative Omicron cases in children and hepatitis cases was demonstrated by Spearman correlation analysis in hepatitis-detected countries. Nonetheless, the absolute number of cumulative Omicron cases is not a positive indicator compared to the cumulative incidence. The cumulative incidence of Omicron cases in children from countries with and without hepatitis detection was not significantly different. Therefore, it is hypothesized that acute hepatitis may be triggered by adenovirus, with SARS-CoV-2 acting as a cofactor that facilitates the progression of adenovirus infection to acute hepatitis (Yi et al., 2022).

There may be a link between the emergence of Omicron and acute hepatitis of unknown origin in children. Nonetheless, further studies are needed to validate this hypothesis.

### 8.3 Adeno-associated virus 2 with helper virus coinfection

In July, two non-peer-reviewed studies from the United Kingdom provided new insights into the possible etiology of the disease: children may be infected with both viruses and harbor genes that lead to an overactive immune response. In addition to the many studies that have reported the detection of adenovirus 41 in children with acute hepatitis of unknown origin, these two studies detected another virus, adeno-associated virus 2 (AAV2), in most children and carried a specific HLA genotype (DRB1*04:01).

AAV2 is a human parvovirus that depends on coinfection with helper viruses such as adenovirus or herpesvirus for efficient reproduction. Many people are infected with AAV2 by age 10, although the virus can remain latent in cells until a helper virus activates it.

A study conducted in Glasgow, Scotland, reported nine early cases and 58 controls. NGS and PCR detected AAV2 in plasma samples from nine patients and liver samples from four patients but not in matched control children. In addition, they found a higher prevalence of the class II HLA-DRB1*04:01 allele in eight of nine children (89%) compared to this HLA gene among Scottish blood donors (15.6%). HAdV (species C and F) and human herpesvirus 6B (HHV6B) were detected in 6/9 and 3/9 affected cases, including 3/4 and 2/4 liver biopsies, respectively (Ho et al., 2022).

Another study by a team in London investigated 28 cases of acute hepatitis in children of unknown origin, including liver samples from five children who required transplantation and blood samples from the remaining children who did not. 16 of the 17 samples tested for AAV2 were positive during RNA sequencing of liver samples (Morfopoulou et al., 2022).

These two studies propose a new etiological hypothesis that acute hepatitis in children of unknown origin may result from the interplay between AAV2, helper virus (e.g., adenovirus), and genetic susceptibility (a specific HLA genotype).

Although genetic material from AAV2 was discovered in the liver cells of patients, neither viral proteins nor actual copies of the virus were detected, emphasizing that AAV2 may trigger an immunological response that is harmful to the organ rather than directly harming liver cells.

There were a few limitations associated with these two studies. First, the number of study subjects was small. Second, current evidence only suggests a correlation with AAV2 with no proof of a causal relationship. It is highly conceivable that AAV2 is only a biomarker for coinfection with ADV or even HHV6B. Many scientists are still cautious about the conclusions of these two studies, and further mechanistic studies and large-sample research studies are warranted to confirm them.

### 8.4 Immunodeficiency

Gao, SH. hypothesized that children with severe acute hepatitis may have an underlying immunodeficiency, given that most cases were in children aged five or younger. In addition to immune insufficiency in children of these ages and immunodeficiency due to lack of pathogen exposure in children as a result of COVID-19 prevention and control measures, primary immunodeficiency should also be investigated (Gao et al., 2022).

In July, the Glasgow Research Group revealed that most children (8/9) carried a specific HLA genotype (DRB1*04:01) (Ho et al., 2022), associated with some autoimmune diseases, such as autoimmune hepatitis (AIH) and rheumatoid arthritis. It is well known that AIH can be classified into two subtypes; type 1 and type 2. Approximately two-thirds of pediatric AIH cases are AIH-1, which usually appears in adolescence, whereas AIH-2 affects younger children, including infants. Up to 67% of patients in children present with an acute onset. Fulminant manifestation is more frequent in AIH-2. AIH-1 susceptibility is conferred by MHC class II HLA DRB1*03 at all ages, whereas DRB1*04 is susceptible to late-onset disease; AIH-2 is associated with possession of DRB1*07 and DRB1*03 (Mieli-Vergani et al., 2018; Di Giorgio et al., 2020; Terziroli Beretta-Piccoli et al., 2022). The class II HLA DRB1*0401 allele may represent a disease susceptibility gene, but the local population structure may overestimate the significance (Ho et al., 2022). Indeed, it has been established that HLA serotypes vary across ethnic groups and regions. AIH-1 is closely related to DRB1*0301 and DRB1*0401 alleles in Caucasian adults; DRB1*0405 in Japan and Argentina (Terziroli Beretta-Piccoli et al., 2022). Moreover, DRB1*04 does not increase children’s susceptibility to AIH and even exerts a protective effect compared to adult patients (Gregorio et al., 1997).

Therefore, genetic testing, major histocompatibility complex molecular phenotyping, and immune function testing, including testing for humoral, cellular, and innate immune responses, should be undertaken on these children.

### 8.5 A novel adenovirus/a new variant of SARS-CoV-2/a new variant of another known pathogen/a new pathogen

In addition, acute hepatitis in children of unknown origin is likely caused by the emergence of a novel adenovirus/a new variant of SARS-CoV-2/a new variant of another known pathogen (cytomegalovirus, EBV, respiratory syncytial virus, enterovirus, parainfluenza virus, *etc.*)/a new pathogen with altered characteristics, with or without the cofactors mentioned above.

Suppose it is a new variant of adenovirus. It is of interest whether it is related to the recombination and mutation of adenovirus under selection pressure that may occur after mass vaccination with adenovirus vector vaccines.

There is a lack of data to support this hypothesis, especially when whole-genome sequencing results from multiple cases have not been established. However, the phenomenon of a novel adenovirus/a novel variant of SARS-CoV-2/a novel variant of other known pathogens/a new pathogen arising from naturally occurring genome recombination is highly possible.

### 8.6 A non-infectious cause

Toxicological investigations have revealed no major findings, with no indication of paracetamol, fluconazole, or aflatoxin B1-induced toxicity (UK Health Security Agency, 2022b).

Environmental exposure that may influence adenovirus infection has not been elucidated. According to the UKHSA report, approximately 70% of children affected whose families were interviewed reported recent contact with pet dogs. In the extended investigations, however, there was no indication that dogs played a role in hepatic syndrome, and no public health issues were associated with dog exposure and hepatic syndrome (UK Health Security Agency, 2022a).

With limited studies based on liver biopsy samples, there is little evidence to support direct viral infection of HAdV-F41, SARS-CoV-2, or AAV2 in explanted livers, and neither immunohistochemistry nor proteomic analysis has been used to detect viral proteins. Instead, studies have found evidence of high HLA class II allele expression in transplant cases, which increases the genetic risk of unknown hepatitis in these children (Ho et al., 2022; Morfopoulou et al., 2022). Nevertheless, we cannot completely rule out an immune-mediated phenomenon in susceptible children with prior viral infections. Therefore, the first four hypotheses mentioned above remain the most likely hypotheses.

## 9 Treatment

Treatment of hepatitis depends on the underlying etiology. Due to the lack of clarity surrounding disease etiology, no specific treatment is available. It is vital to administer comprehensive treatment, monitor conditional changes, dynamically assess laboratory indicators, be alert to liver failure, and prevent complications.

On the premise of sufficient evidence to support viral infection, it is recommended to use broad-spectrum antiviral drugs. Cidofovir (CDV) is the mainstay antiviral drug used to treat adenovirus infection. Besides, cidofovir’s lipid ester analog, brincidofovir, may be a promising salvage therapy for treatment in cases of renal toxicity induced by cidofovir or unsatisfactory clinical response (Gu et al., 2021). The nucleoside analog filociclovir (FCV) has recently been reported as a potent inhibitor of HAdV-F41 in cell culture (Tollefson et al., 2022).

Recent investigations revealed that iPALF is characterized by excessive cellular and humoral systemic inflammatory responses (Alonso et al., 2017). Immunosuppressants have been used to treat PALF associated with activated CD8^+^ T cell hepatitis (Squires et al., 2022), including corticosteroids for nonspecific immunosuppression, and anti-thymocyte globulin, cyclosporine, or etoposide for more specific suppression of T cell responses. Anti-cytokine therapy may also play a potential role (Alonso et al., 2017). However, the risks and benefits of immunosuppressive therapy should be weighed.

Interestingly, it has been shown that plasmapheresis can improve short-term survival and combined with other extracorporeal therapies, it can serve as a bridge to transplantation (Squires et al., 2022). For children with poor response to active comprehensive medical treatment, liver transplantation should be considered if there is no contraindication.

## 10 Prevention and control

Until the etiology is further understood, the WHO recommends implementing general infection prevention and control (IPC) measures, including regular hand hygiene, wearing a well-fitted mask, social distancing, covering coughs and sneezes and avoiding touching eyes, nose or mouth, ensuring adequate ventilation when indoors, and attention to food and water hygiene (World Health Organization, 2022b). Health facilities should adhere to standard precautions for suspected or probable cases and undertake contact and droplet precautions (World Health Organization, 2022b).

## 11 Conclusion

In summary, acute hepatitis of unknown origin in children cannot be attributed to a single cause and is most likely caused by a combination of factors. Combined with the previous hypothesis, we speculate that for a subset of children with the class II HLA allele HLA-DRB1*04:01, previous infection with SARS-CoV-2 leads to superantigen-mediated immune activation. In addition, secondary ADV infection and coinfection or reactivated hepatotropic AAV2 infection lead to abnormal host response or susceptibility, resulting in severe immune dysregulation and liver inflammation. Nevertheless, further data and research are required to comprehend the specific mechanism fully.

In addition, the heterogeneous case definitions used by different organizations/countries increase the challenge of data collection systems and comparisons between data. Indeed, it is better to establish a uniform case definition to facilitate data statistics and analysis to ensure that better and more accurate conclusions can be drawn. The WHO has established a clinical Case Report Form and urges countries to participate in the Global Clinical Platform for all cases meeting the WHO case definition. A standardized global clinical data analysis can aid in comprehending the disease’s etiology, clinical manifestations, natural course, and severity and can better guide the public health response and establish clinical management guidelines, including investigation methodologies and interventions for infection prevention and control (World Health Organization, 2022b). Most importantly, clinicians should collaborate with researchers such as immunologists and epidemiologists to study the epidemiology and pathogenesis of this disease. For children with acute hepatitis of unknown etiology but serum transaminases (ALT, AST) below 500 U/L, close attention should also be paid to their disease progression. If a mechanism associated with a postinfectious inflammatory process is identified, early administration of corticosteroids or immunomodulators may prevent progression to acute liver failure (Alexander and Deep, 2022).
